# CXCR4-targeted therapy in lung cancer: plerixafor as a promising antimetastatic agent

**DOI:** 10.3389/fphar.2025.1683585

**Published:** 2025-11-26

**Authors:** Prasanna Srinivasan Ramalingam, Muhammad Afzal, M. Arockia Babu, Manjunath Mirle Rekha, Samir Sahoo, Surya Nath Pandey, Kavita Goyal, Md Sadique Hussain, Gaurav Gupta, Purushothaman Balakrishnan, Sivakumar Arumugam

**Affiliations:** 1 Protein Engineering Lab, School of Biosciences and Technology, Vellore Institute of Technology, Vellore, Tamil Nadu, India; 2 Department of Pharmaceutical Sciences, Pharmacy Program, Batterjee Medical College, Jeddah, Saudi Arabia; 3 Institute of Pharmaceutical Research, GLA University, Mathura, Uttar Pradesh, India; 4 Department of Chemistry and Biochemistry, School of Sciences, JAIN (Deemed to Be University), Bengaluru, Karnataka, India; 5 Deptartment of General Medicine, IMS and SUM Hospital, Siksha ‘O’ Anusandhan (Deemed to be University), Bhubaneswar, Odisha, India; 6 Deptartment of Pharmacology, Teerthanker Mahaveer College of Pharmacy, Teerthanker Mahaveer University, Moradabad, Uttar Pradesh, India; 7 Sharda University, Greater Noida, India; 8 Uttaranchal Institute of Pharmaceutical Sciences, Uttaranchal University, Dehradun, Uttarakhand, India; 9 Centre for Research Impact and Outcome-Chitkara College of Pharmacy, Chitkara University, Rajpura, Punjab, India; 10 Centre of Medical and Bio-allied Health Sciences Research, Ajman University, Ajman, United Arab Emirates; 11 TanBio R and D Solution, Thiruvarur, Tamilnadu, India

**Keywords:** CXCR4, lung cancer, tumor progression, plerixafor, metastasis, prognosis, immune cell migration, tumor microenvironment (TME)

## Abstract

Metastasis remains the prime cause of poor prognosis in lung cancer, a leading cause of cancer-related mortality worldwide. Because CXCR4/CXCL12 constitutes a powerful therapeutic target to counter tumor progression, immune evasion, and therapy resistance, it plays a pivotal role in lung cancer. Expression of CXCR4 is high in non-small cell lung cancer (NSCLC) and small cell lung cancer (SCLC) and has been correlated with aggressive tumor behavior increased metastatic spread to the bone marrow, the liver, and the brain, and poor overall survival. Studies in preclinical models have demonstrated that plerixafor is a CXCR4 inhibitor that can reduce tumor cell migration, increase chemosensitivity, and re-establish immune response to limit metastasis and increase treatment efficacy. Furthermore, clinical trials combining plerixafor with chemotherapy as well as immune checkpoint inhibitors in NSCLC patients demonstrate that this drug increases T cell infiltration, increases the ability of the tumor to stimulate anti-tumor immunity, and increases progression-free survival. However, although there are promising preclinical and encouraging early clinical data, it is important to address several issues before CXCR4-targeted therapies can become an integral part of lung cancer treatment. They include tumor heterogeneity, adaptive resistance mechanisms, as well as the complexity in the tumor microenvironment of CXCR4 signaling. Additionally, drug development strategies aimed at suppressing CXCR4-driven immune suppression and radioresistance must be combined with chemotherapy, radiotherapy, and immunotherapy therapies to maximize therapeutic benefits. Imaging of CXCR4 with specific PET and the selection of patients on CXCR4 biomarker criteria offer the possibility of further improving precision medicine approaches so that CXCR4-targeted therapies will only be given to the most CXCR4-responsive patients. The role of CXCR4 in lung cancer pathogenesis and development is critically reviewed, the most recent results on plerixafor inhibition of CXCR4 are summarized, and new, potential strategies for combination treatment of CXCR4 with other inhibitors are explored.

## 1 Introduction

Despite a significant decrease in mortality from this disease, lung cancer continues to be the primary cause of cancer-related death worldwide, recording approximately 2.2 million new cases and 1.9 million deaths annually (Sharma, 2022). Despite these recent advancements in targeted therapy, immunotherapy, and chemotherapy, there is still high resistance to lung cancer therapeutics (Saha et al., 2024), and survival is dramatically compromised by the development of adaptive ways for the tumor to progress and metastasize (Li C. et al., 2023; Kuang et al., 2024). Non-small cell lung cancer (NSCLC), which comprises 85% of lung cancer cases (Wang N. et al., 2023), has a 5-year survival rate < 20% and small cell lung cancer (SCLC) which is a more aggressive subtype is less fortunate with its poor prognosis because it is rapidly disseminated via early metastatic spread (Megyesfalvi et al., 2023). Almost half of lung cancer cases are identified at advanced stages, at which point curative intervention is limited and metastasis is the predominant barrier to long-term survival (Altorki et al., 2019). Metastatic sites are most common in the brain, liver, bones, and adrenal glands, and median survival for individuals with metastatic lung cancer is typically less than 1 year, even with treatment (Pass et al., 2012; Zhou et al., 2021). Despite previous clinical benefits in immune checkpoint inhibitors (ICIs) and tyrosine kinase inhibitors (TKIs) and a high incidence of disease progression despite therapy, there is an urgent need for novel therapeutics directed towards metastatic pathways (Gravina et al., 2024).

The CXCR4/CXCL12 chemokine signaling is among the most well-characterized mechanisms whereby tumors undergo metastasis and therefore facilitate increased migration, immune evasion, and treatment resistance (Morein et al., 2020; Britton et al., 2021). CXCR4, a GPC receptor, binds its ligand CXCL12 (stromal cell-derived factor 1, SDF1), which is highly expressed in metastatic target organs (Hang, 2020). In physiological conditions, CXCR4 controls immune cell trafficking, stem cell homing, and tissue repair (Miller et al., 2008). However, in lung cancer, CXCR4 overexpression facilitates tumor cells to migrate along chemotactic gradients of its ligand CXCL12 (stromal cell-derived factor 1, SDF-1) toward CXCL12-enriched metastatic niches, where tumor adhesion, survival, and immune escape are enhanced (Chandra et al., 2021). Stimulation of the CXCR4-CXCL12 cascade activates oncogenic pathways (MAPK/ERK, PI3K/AKT, PLCβ/Ca^2+^, and SRC/FAK) that promote epithelial-to-mesenchymal transition (EMT), angiogenesis, and are central to therapy resistance (Wang et al., 2021; Yang et al., 2023). This cascade allows cancer cells to detach from the primary tumor, circulate via the bloodstream, and colonize in distant organs where these tumor cells can establish themselves into a secondary tumor (Panda et al., 2016; Mir et al., 2021). Furthermore, CXCR4 helps in immunosuppression by driving regulatory T cells (Tregs), myeloid-derived suppressor cells (MDSCs), and tumor-associated macrophage (TAMs) into the tumor microenvironment (TME) along with each other to create an immunosuppressive TME (Hussain et al., 2025) and at the same time neutralize chemotherapy, radiotherapy, and ICIs therapies (Barnestein et al., 2022; Wu et al., 2025). Since CXCR4 is pivotal in the advancement of lung cancer, focusing on this cascade is an ideal treatment strategy (Yang et al., 2020). Preventing tumor cell migration, limiting metastatic burden, and enhancing anti-tumor immune activities to overcome resistance to standard therapies (Peng et al., 2025), blocking CXCR4 signaling is the way (Eckert et al., 2018). The small molecule antagonists, monoclonal antibodies, and peptide inhibitors for CXCR4 have been explored for their ability to inhibit CXCR4 (Zhou et al., 2019). Currently, there existing lung cancer treatments mainly target inhibiting the primary tumor progression growth and managing the micrometastases (Wood et al., 2014). Despite that, none directly attack CXCR4-driven metastatic cascade (Ko et al., 2021).

On the preclinical level, CXCR4 inhibition has been demonstrated to significantly reduce lung tumor growth, increase chemotherapy and radiotherapy sensitivity, and also to improve immune infiltration in the TME (D’Alterio et al., 2019). Furthermore, it has been noted that pharmacological inhibition of CXCR4 also potentiates the effect of ICIs, including anti-PD-1 and PD-L1, in reversing tumor-induced immunosuppression (Park et al., 2022). There are several inhibitors of CXCR4 under investigation (Fujiwara et al., 2020). Plerixafor was originally developed to mobilize hematopoietic stem cells and is also shown to exert an anti-metastatic and immunomodulatory effect in preclinical lung cancer models (Regan, 2017). Other CXCR4 antagonists, balixafortide, mavorixafor, and LY2510924 are being evaluated to enhance combination therapies (Leo and Sabatino, 2022). In contrast to standard epidermal growth factor receptor (EGFR)-TKIs and chemotherapy, which mainly inhibit primary tumor growth, CXCR4 inhibitors exclusively prevent metastatic seeding, escape from the immune system, and therapeutic resistance, which should be crucially incorporated into current lung cancer treatment regimens (Otsuka, 2011; Alqudah et al., 2025). In a study, it was found that lung cancer can be made more sensitive to drugs with the help of blocking CXCR4 (Su et al., 2005). Eventually, EGFR inhibitors or chemotherapy fail in most NSCLC patients, and tumors can evolve resistance and start increasing in number again (Choi et al., 2012). Nevertheless, blockade of CXCR4 has been shown to inhibit these adaptive resistance pathways and thus could prolong the therapeutic benefit of the standard treatments (Chaudary et al., 2021). For the clinical benefits of CXCR4 targeted therapy in lung cancer management, the patient selection strategies, combination therapy approaches, and optimized drug formulations will be moving forward (Shojaei et al., 2024). Clinical efficacy, safety profile, and optimal treatment strategies of CXCR4 inhibitors in monotherapy and combination with chemotherapy, immunotherapy, and radiotherapy are being continuously evaluated as monotherapy and in clinical trials as combination therapy (Sabir et al., 2021; Alexandru et al., 2024). In this review, we explore the function of the CXCR4/CXCL12 axis in lung cancer metastasis and its therapeutic potential for stopping tumor progression and strengthening cancer treatment.

## 2 CXCR4/CXCL12 axis in lung cancer

### 2.1 Role of CXCR4 in tumor growth and metastasis

The development of these effector functions of lung cancer has been linked to the function of the CXCR4/CXCL12 axis (Mezzapelle et al., 2022). The chemokine CXCL12, which is highly expressed in metastatic niches, including the bone marrow, liver, and the brain, has its receptor CXCR4 that engages in a signaling cascade that stimulates tumor proliferation, survival, and resistance to apoptosis through PI3K/AKT, MAPK/ERK, and JAK/STAT pathways (Mousavi, 2020). The CXCR4/CXCL12 axis increases metastasis in lung cancer by mediating chemotaxis of CXCR4+ tumor cells against CXCL12-rich secondary sites (bone marrow, liver, brain), which triggers PI3K/AKT, MAPK/ERK, and JAK/STAT signaling and EMT, invasion, and intravasation/extravasation, and establishes an immunosuppressive TME through Tregs, MDSCs, and TAMs recruitment (Wang et al., 2016). Furthermore, the recruitment of these to a TME via CXCR4 further contributes to systemic immunosuppression through the release of cytokines, like IL-10 and TGF-β, which prevents cytotoxic T cell killing capacity (Nengroo et al., 2022b). This axis plays a functional role in every stage of the metastatic cascade, including local invasion and vascular dissemination and colonization and outgrowth at the new organs, as well as in resistance to therapy during chemotherapy, radiotherapy, and immune checkpoint blockade.

Elevated CXCR4 expression in lung cancer has been correlated with enhanced tumor aggressiveness as well as a poor prognosis in tandem with both enhanced therapy resistance to chemotherapy and ICI therapy (Tajaldini et al., 2023). CXCR4-expressing lung cancer cells are more metastatic because the receptor promotes chemotactic migration towards CXCL12-enriched sites, allowing invasion, extravasation, and colonization (Sun et al., 2010). In addition, CXCR4 signaling helps to bring about the critical EMT process, which promotes gaining stem-like characteristics, the ability to be more motile, and greater resistance to being killed by apoptosis, leading to more aggressive and efficient metastasis (Sabbah et al., 2008; Nantajit et al., 2015).

The importance of CXCR4 to metastasis is also potentiated by its contribution to formulating the TME into an immunoevasive environment (López-Gil et al., 2021; Wang Z. B. et al., 2024). CXCR4 signaling recruits TAMs, MDSCs, and Tregs, collectively suppressing anti-tumor immunity and promoting therapy resistance (Kohli et al., 2022). Liu et al. demonstrated that while CXCR4 is expressed in both normal and tumor lung tissues, CXCR7 (presently ACKR3), a functionally related receptor, is exclusively upregulated in tumors, further promoting migration, invasion, and metastasis to the liver and bone marrow (Liu et al., 2020). This indicates that CXCR7 may act as an alternative driver of metastasis, potentially compensating for CXCR4 inhibition. Similarly, Xing et al. reported that lidocaine, a local anesthetic, inhibits CXCR4-mediated migration in NSCLC by disrupting CXCL12-induced cytoskeletal remodeling, reducing intracellular Ca^2+^ release, and altering actin polymerization (Xing et al., 2022). These findings suggest that targeting CXCR4 may not only disrupt metastasis but also create new therapeutic opportunities using repurposed drugs. The therapeutic targeting of CXCR4 is of great interest since this axis plays a key part in the development of lung cancer. Inhibiting the tumor cell’s movement towards the CXCR4 prevents tumor cell migration and immune suppression and promotes the enhancement of the efficacy of chemotherapy and immunotherapy. Despite these challenges, we nonetheless face therapy resistance, compensatory signaling via CXCR7, and potential off-target effects.

CXCL12 plays a nuclear role in tumor-intrinsic EMT, invasion, survivability, and metastatic seeding mediated by tumor intrinsic CXCR4 signaling (through PI3K/AKT, MAPK/ERK, JAK/STAT) as well as CXCL12 chemotaxis induced by microenvironmental CXCL12 in stromal and endothelial cells. In combination, these intrinsic and extrinsic inputs coordinatively organize the entire metastatic cascade and therapy resistance of lung cancer.

### 2.2 CXCR4 and the tumor microenvironment (TME)

Interaction between cancerous cells, stromal components, and immune cells in the lung cancer TME requires the key regulator of this process, CXCR4 (Santagata et al., 2021). Furthermore, fibroblast activation induced by CXCR4 induces a desmoplastic reaction with dense stromal barriers to suppress immune infiltration and drug penetration, which is correlated with low response to chemotherapy, radiotherapy, and immunotherapy (Monteran and Erez, 2019; Li X. P. et al., 2023). For the step-wise metastatic mechanism driven by CXCR4/CXCL12 in lung cancer, see Section 2.1.

CXCR4 expression in lung cancer has been related to the enrichment of lung CSC with a capacity for self-renewal, metastasis, and resistance to treatment. Moreover, hypoxic conditions in TME stimulate tumor aggressiveness driven by CXCR4, due to hypoxia-inducible factor 1 alpha (HIF1α) stabilization of CXCR4 expression and tumor cell survival in nutrient deprivations (Miranda-Galvis and Teng, 2020; Emami Nejad et al., 2021). Investigations have shown the critical function of CXCR4 in these processes, with Jäger et al. reporting that CXCR4-overexpressing NSCLC cells exhibit increased tumorsphere formation and EMT, partially mediated by macrophage migration inhibitory factor (MIF) and IL-6 signaling, which drive tumor progression and enhance stromal support (Jäger et al., 2020). Similarly, Andtbacka et al. showed that mavorixafor, a CXCR4 inhibitor, enhances immune infiltration in tumors by increasing antigen presentation, CD8^+^ T-cell activity, and IFN-γ expression, with combination therapy improving responses to ICIs in solid tumors, including NSCLC (Andtbacka et al., 2022). These outcomes suggest that CXCR4-targeted therapies not only disrupt cancer cell migration and invasion but may also enhance immune-mediated tumor clearance, particularly in combination with immunotherapy. The practical application of targeting this pathway lies in its central role in shaping an immunosuppressive and therapy-resistant TME that makes overcoming resistance mechanisms and increasing therapeutic efficacy a very promising approach. Nevertheless, owing to the complexity of CXCR4 interaction in the TME, combinatorial treatment of CXCR4 with chemotherapy, immunotherapy, or stromal targeting agents is required to achieve maximum therapeutic effect for lung cancer patients.

The CXCR4/CXCL12 axis also communicates with immune checkpoints (from the mobilization of myeloid/lymphoid cells and predictability of immune resistance to immunoprotective T-cell exclusion) and also with hematopoietic trafficking, in which CXCR4 blockade mobilizes myeloid/lymphoid cells but may not disrupt pulmonary host-defense dynamics, which is pertinent both in combination with ICIs and safety monitoring during on-treatment.

### 2.3 Clinical significance of CXCR4 expression in lung cancer

CXCR4 overexpression correlates with poor prognosis, greater metastatic potential, and resistance to therapy in NSCLC and SCLC (Zhang et al., 2015). Mechanistic underpinnings of CXCR4-driven metastasis are summarized in Section 2.1; here, we focus on its prognostic and biomarker implications and on patient selection.

CXCR4 expression is upregulated in NSCLC (Su et al., 2005) and reported in SCLC models/cohorts, with higher levels in advanced/metastatic disease. Individuals with CXCR4 overexpression have reduced overall survival (OS) and disease-free survival (DFS), and it seems that the high CXCR4 expression has a negative influence, especially in stage III and IV lung cancer, correlated with metastasis to the brain, liver, and bone (Franco et al., 2012; Pulido et al., 2017). Moreover, CTCs positive for CXCR4 are also associated with a poorer prognosis and are a predictive and prognostic biomarker. Non-invasive detection of tumors expressing CXCR4 has been accomplished by imaging modalities using radiolabelled antagonists of CXCR4 (Oriuchi et al., 2020). Lakhanpal et al. demonstrated the potential of 68Ga-plerixafor PET/CT for visualizing CXCR4-expressing malignancies, with a strong correlation between PET signal and 18F-FDG uptake, confirming its diagnostic utility (Lakhanpal et al., 2023). Similarly, Dreher et al. showed that [68Ga]Ga-Pentixafor PET/CT imaging detected CXCR4 overexpression in SCLC and other solid tumors, suggesting its role in guiding targeted therapy selection for patients with CXCR4-enriched tumors (Dreher et al., 2024).

Beyond its prognostic and diagnostic uses, the increase of CXCR4 expression is a critical driver of therapy resistance by regulating DNA damage repair, cancer stemness, and immune evasion, all of which impair response to chemotherapy, radiotherapy, and ICIs. Many strategies have been explored blocking CXCR4 signaling to improve chemoresponsive and immunotherapeutic responses, and there have been promising results from different preclinical and clinical studies (Gibson and Davids, 2015; Wang S. et al., 2024). Weiss et al. investigated 64Cu-AMD3100, a radiolabelled CXCR4 antagonist, and demonstrated high tumor selectivity and strong tumor-to-muscle and tumor-to-blood ratios, making it a viable option for non-invasive quantification of CXCR4 expression to inform treatment decisions (Weiss et al., 2012). In a similar approach, Azad et al. evaluated 89Zr-labeled CXCR4 monoclonal antibody (89Zr-CXCR4-mAb) imaging in NSCLC models and revealed enhanced PET uptake in tumors with high CXCR4 expression, with therapeutic responses correlating with CXCR4 levels, supporting its role in precision medicine approaches for lung cancer (Azad et al., 2016). Therefore, a growing interest in the therapeutic and biomarker utility of CXCR4 for patient selection in lung cancer is motivated by the strong evidence that implicates it in lung cancer progression and therapy resistance. Therefore, integration of histology-aware CXCR4 imaging and biomarker strategies may enable more precise treatment strategies to not only guide clinicians to identify patients with the highest chances of response to CXCR4-directed therapies such as plerixafor, balixafortide, and motixafortide but also address treatment resistance through targeted combination approaches.

The expression of CXCR4 is different across lesions and over time in patients and in the same patient. Hypoxia/HIF-1α can upregulate CXCR4 in regional niches, CXCR7 can overcome CXCR4 blockade in relationship tumors and add biological variation, which can quench local reactions. Based on this, lesion-level measurement by CXCR4-targeted PET and/or tissue biomarkers may aid in identifying patients with CXCR4-high disease and inform response-adaptive treatment.

Clinically-trial CXCR4 assays should be aforementioned with regarding clinical-trial (PET) standards, state acquisition/reconstruction; in tissue IHC state clone, scoring method, and inter-observer validations, on blood assays include platform and repeatability. Cutoffs of eligibility must be set, such as lesion level uptake of PET above some reference level or a top quantile cutoff, and/or tissue CXCR4 levels above a prespecified score; heterogeneity between sites may be treated by the requirement that there be at least one target lesion that satisfies PET requirements, and the variance of locations is obviated by recording. PET conventions and/or other biomarker dynamics can be used as a basis for on-treatment assessment that can provide response-adaptive therapy. The following steps operationalize selection and accept non-homogeneous CXCR4 expression as earlier explained.

## 3 Plerixafor: a CXCR4 antagonist in lung cancer therapy

### 3.1 Mechanism of action

**FIGURE 1:**
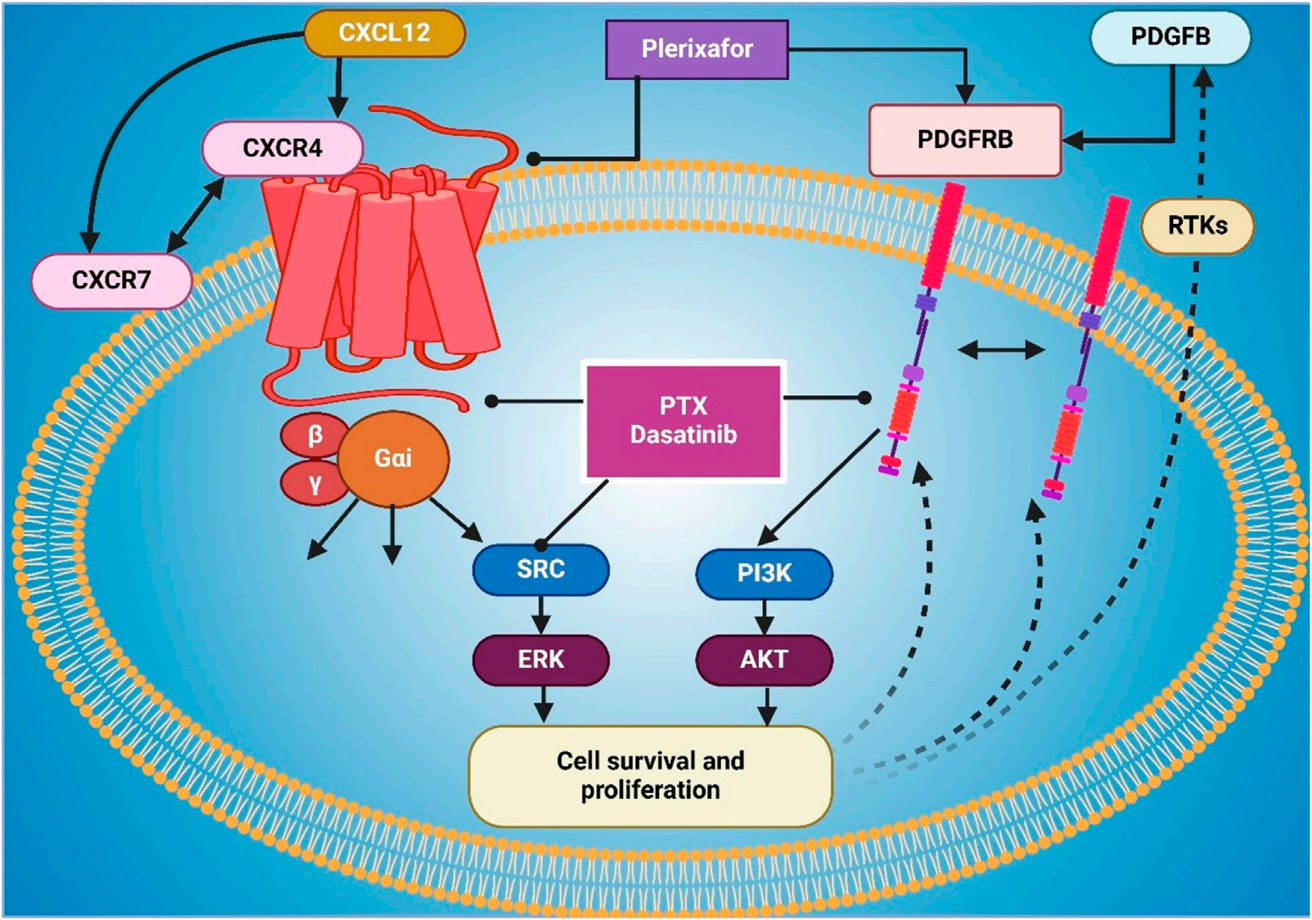
The figure illustrates the CXCR4 signaling pathway and its role in cell survival and proliferation, highlighting the impact of inhibitors such as Plerixafor and Dasatinib. CXCL12 binds to CXCR4, activating downstream signaling through Gαi proteins, which subsequently stimulate SRC-ERK and PI3K-AKT pathways, promoting cell survival and proliferation. CXCR7 also contributes to CXCR4 activation. Plerixafor inhibits CXCR4 by blocking CXCL12 binding, while Dasatinib and PTX disrupt SRC and PI3K signaling. Additionally, PDGFB activates PDGFRB and receptor tyrosine kinases (RTKs), which interact with CXCR4 signaling to enhance survival pathways.

AMD3100 (plerixafor), a selective CXCR4 antagonist and disrupts the CXCR4/CXCL12 signaling and has shown antitumor activity in preclinical lung cancer models (Wang et al., 2016). CXCR4 receptor binds its ligand, CXCL12 (SDF-1), which is overexpressed in lung cancer cells, and the interaction of this receptor-ligand drives tumor proliferation, migration, and therapy resistance (Cojoc et al., 2013). Plerixafor prevents key oncogenic processes such as cancer cell mobilization, TME interactions, and immune suppression by blocking CXCR4 binding to CXCL12 (Zhao et al., 2022). Plerixafor reduces migration and metastatic seeding in preclinical systems by blocking CXCR4 (Wang et al., 2014). CXCL12 is additionally secreted by stromal fibroblasts, endothelial cells, and immune cells in lung cancer in such amounts that they form chemotactic gradients drawing CXCR4-expressing cancer cells to metastatic niches such as liver, brain, and bone marrow (Mir et al., 2023; Thapa et al., 2023). Concerning cancer metastasis, plerixafor blocks this chemotactic signaling, which reduces the chance that cancer cells will home to distant organs (Nasrollahzadeh et al., 2020; Lalić et al., 2024). Figure 1 illustrates CXCR4 signaling, its regulation by plerixafor and Dasatinib, and its role in cell survival. Furthermore, plerixafor interferes with the tumor stroma interactions that are known to be responsible for resistance to therapy (Eulberg et al., 2022). It is known that CXCR4 allows cancer stem cell (CSC) survival and immune evasion within the TME (Dzobo et al., 2020).

When used in combination, plerixafor decreases CSC self-renewal and increases sensitivity to chemotherapy and radiotherapy in CSC by blocking CXCR4 (Huang et al., 2019). Additionally, it inhibits the recruitment of immunosuppressive cells to the TME and contributes to immune evasion via an immune-evasive TME (Shao et al., 2022). It potentiates anti-tumor immune response and increases tumor responsiveness to ICIs (Zhang et al., 2022). Finally, plerixafor has been noted to sensitize lung cancer cells to chemotherapy and radiotherapy in the setting of decreased DNA damage repair and survival signaling (Sun, 2016). Plerixafor and cisplatin killed lung cancer cells more and caused reduced tumor growth in preclinical studies (Langhammer, 2013). Furthermore, plerixafor decreases HIF1α, a downstream key force in cancer adaptation to hypoxic conditions, and decreases cancer cell survival (Mortezaee, 2020). The mechanism of action of plerixafor is the inhibition of metastasis by abrogating CXCR4-CXCL12 interactions, the disruption of the immunosuppressive TME, preserving chemosensitivity, and fighting therapy resistance (Russo and Nastasi, 2022). Given these multifaceted effects, a combination of PTG in standard chemotherapy, radiotherapy, and immunotherapy is a promising therapeutic agent for lung cancer (Wang H. et al., 2023), as shown in Figure 1.

### 3.2 Preclinical and clinical studies on plerixafor

Extensive preclinical lung cancer studies have been performed on plerixafor, including inhibiting tumor growth, reducing metastasis, and having synergy with chemotherapy and immunotherapy (Steeg, 2016). Plerixafor exerts significant tumor burden reduction and metastatic spread inhibition to the brain, liver, and bone marrow, common sites of NSCLC dissemination, in murine models of NSCLC (Mishan et al., 2016; Yang et al., 2023). Studies of plerixafor treatment on CXCR4 overexpressing NSCLC cell lines have demonstrated reduced cell migration, inhibited invasion, and enhanced apoptosis, especially in hypoxic conditions where CXCR4 expression is increased (Li C. et al., 2022). Li and Oupicky, investigated biodegradable polymeric plerixafor (PAMD) and found that biodegradable PAMD significantly inhibited cancer invasion and metastasis *in vivo*, suggesting potential as an optimized CXCR4-targeting strategy (Li and Oupický, 2014; Guo et al., 2025).

In combination with chemotherapy, plerixafor also disrupts tumor-stroma interactions and reduces CSC survival in further studies. CXCR4 inhibition is also shown to increase tumor-infiltrating T cells and decrease immunosuppressive MDSCs in preclinical findings, and results in sensitization of tumors to the chemotherapy agents of cisplatin, paclitaxel, and gemcitabine (Xun et al., 2020). Additionally, Plerixafor has proved able to overcome radiation resistance through the inhibition of CXCR4-induced DNA damage repair pathways, leading to more radiosensitive tumors (Eckert et al., 2018). Ko et al. demonstrated that plerixafor-functionalized nanomaterials significantly improved drug delivery to CXCR4-overexpressing tumors, with enhanced tumor accumulation and photothermal anticancer efficacy, suggesting potential applications in CXCR4-targeted nanotherapy (Ko et al., 2018).

Clinical trial research with Plerixafor has been encouraged by preclinical data, and studies of plerixafor’s potential in treating lung cancer have focused on the utilization of plerixafor in combination with chemotherapy and immunotherapy (Bao et al., 2023). Plerixafor has an acceptable safety profile when administered as an early therapy and was found to be capable of mobilizing tumor cells from the bioprotected niches in the bone marrow as well as the TME at a site that is known to limit their vulnerability to treatment (Morland et al., 2020). Among other things, a Phase I trial of plerixafor with chemotherapy in advanced NSCLC was able to show improved response rates as well as prolonged DFS in patients with high CXCR4-expressing tumors (Nengroo et al., 2022a). Further, trial data combining plerixafor with ICIs shows evidence of CXCR4 inhibition improving T cell infiltration, leading to increased immune activation and thus, the immune response rate to treatment in NSCLC (Zhu et al., 2023). Weiss et al. utilized PET imaging with 64Cu-plerixafor to examine CXCR4 expression in solid tumors, showing high CXCR4 levels in metastatic lung adenocarcinomas, further supporting the use of CXCR4-directed therapies in aggressive tumors (Weiss et al., 2017).

Additional research has explored alternative applications of Plerixafor beyond lung cancer, particularly in hematopoietic cell mobilization and fibrosis treatment (MacLean et al., 2024). Pillay et al. investigated plerixafor’s effects on neutrophil mobilization, demonstrating that while it increases circulating neutrophils, it does not impair lung neutrophil dynamics, suggesting minimal impact on respiratory host defense (Pillay et al., 2020). Similarly, Qi et al. analyzed single-cell RNA sequencing data in fibrosis models, showing that plerixafor-mediated CXCR4 inhibition significantly reduced fibrosis progression, highlighting its potential for drug repurposing in fibrosis management (Qi et al., 2024). Devi et al. further investigated CXCR4–CXCL12 signaling in neutrophil homeostasis, revealing that plerixafor enhances circulating neutrophils by preventing their return to the bone marrow, providing insight into its role in neutropenia management (Devi et al., 2013). Furthermore, Lakhanpal et al. optimized radiolabeled plerixafor (plerixafor-DTPA, plerixafor-NOTA) with 68Ga and 177Lu for PET/CT imaging and targeted therapy, showing that 68Ga-plerixafor PET/CT successfully identified CXCR4-expressing lung lesions, correlating with 18F-FDG uptake, while 177Lu-plerixafor exhibited high CXCR4 binding affinity and cytotoxicity in lung cancer cells, emphasizing its potential as a theranostic agent (Lakhanpal et al., 2022). In contrast, Blayney et al. evaluated Plinabulin as an alternative mobilizing agent, finding that it effectively mobilizes CD34^+^ hematopoietic cells without significantly inhibiting CXCR4, making it a viable option for NSCLC patients unresponsive to G-CSF-based mobilization (Blayney et al., 2018). Plerixafor, as a CXCR4 targeted treatment for lung cancer, is well supported by preclinical or early clinical evidence as a potential therapeutics for lung cancer in combination with chemotherapy, radiotherapy, and immunotherapy. Thus, it is a promising agent for lung cancer therapy because of its ability to enhance immune response, suppress therapy resistance, and accelerate targeted drug delivery. Further trials will confirm its clinical efficacy and long-term therapeutic potential and establish the way for widespread clinical adoption in CXCR4-driven malignancies.

### 3.3 Plerixafor in combination therapy

**FIGURE 2:**
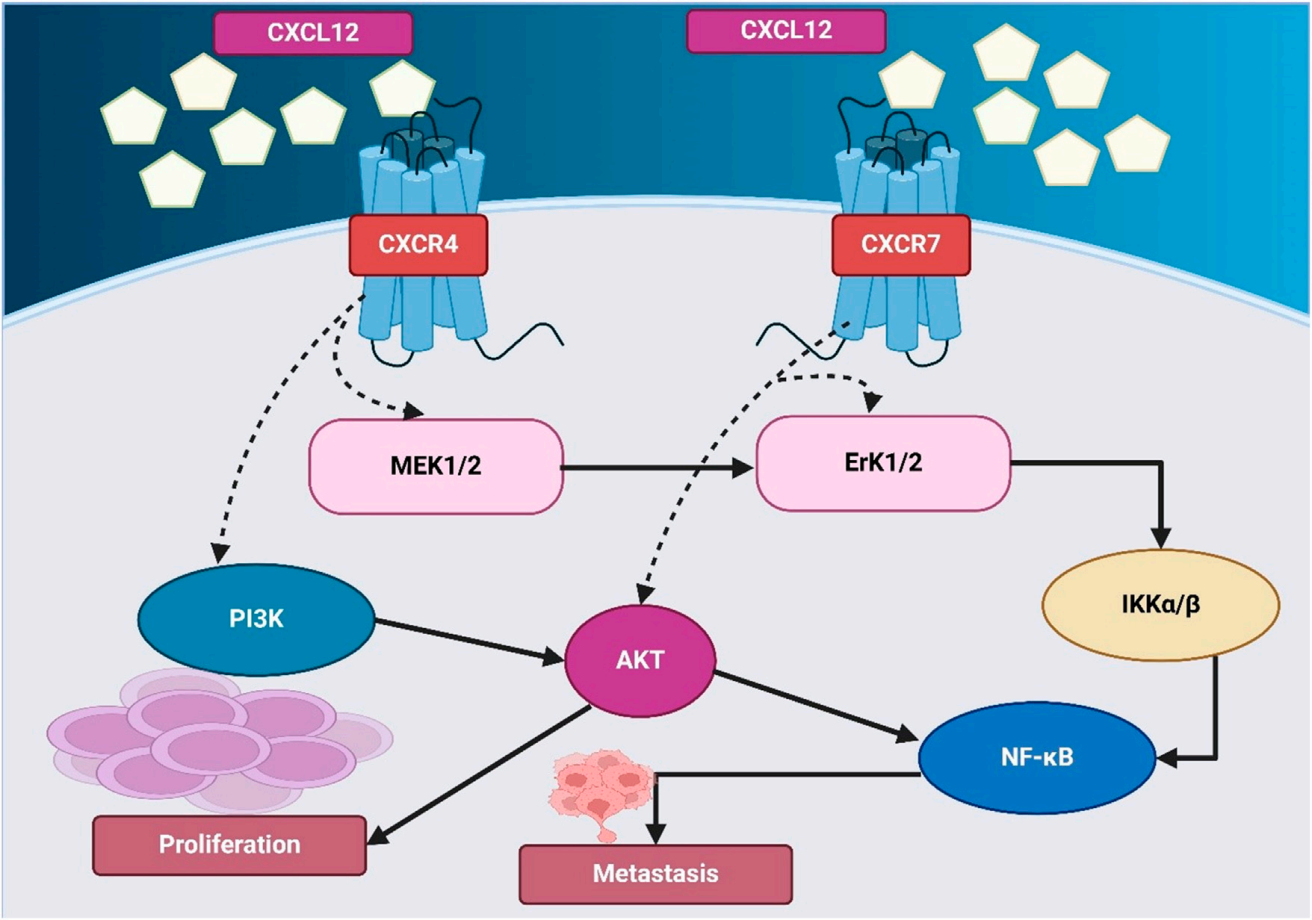
The figure illustrates the CXCL12-mediated signaling pathways through CXCR4 and CXCR7, driving proliferation and metastasis. CXCL12 binds to CXCR4 and CXCR7, activating downstream signaling cascades. CXCR4 signaling involves MEK1/2 and PI3K, leading to AKT activation, which promotes cell proliferation. CXCR7 signaling activates ERK1/2, which in turn stimulates IKKα/β and NF-κB, contributing to metastasis. AKT also directly regulates NF-κB, further enhancing metastatic progression.

Plerixafor, as a CXCR4 antagonist, has significantly more efficacy in mobilizing and maintaining CD34^+^ cells and cells for cancer immunotherapy, chemotherapy, and radiotherapy when exacerbated with any standard cancer treatment, including chemotherapy, immunotherapy, radiotherapy, or a combination of all (Crees et al., 2023). Plerixafor blocks CXCR4 and enables tumor cells to be mobilized from protective niches like the bone marrow and hypoxic tumor regions and making them more susceptible to therapeutic agents (Cancilla et al., 2020). Across preclinical models and early human signals in small cohorts, adding plerixafor to chemotherapy has been associated with greater tumor control and immune infiltration; however, definitive clinical benefit in lung cancer has not been established (Chaudary et al., 2024; Thapa et al., 2024). Figure 2 shows CXCL12-CXCR4/CXCR7 signaling, activating AKT and NF-κB to drive proliferation and metastasis. To aid navigation, key plerixafor-based combination strategies and outcomes are summarized in Table 1.

**TABLE 1:** Summary of plerixafor-based combination strategies in lung cancer models and translational studies.

Combination/Strategy	Model or setting	Key outcome	Mechanistic note	Reference
Plerixafor + chemotherapy (cisplatin, paclitaxel, gemcitabine)	NSCLC preclinical models	Greater tumor regression and fewer metastases vs. chemo alone; ↑T-cell infiltration; ↓MDSCs	CXCR4 blockade disrupts stromal protection and CSC maintenance	Xue et al. (2021)
Plerixafor + RT	Preclinical NSCLC	Enhanced radiosensitivity; ↑tumor cell death post-RT	Inhibits CXCR4-linked DNA repair pathways; overcomes hypoxia-associated radioresistance	Langhammer (2013)
Plerixafor + ICI (anti-PD-1/PD-L1)	Preclinical and early clinical NSCLC	↑T-cell infiltration; reversal of immune exclusion; improved responses	Remodels TME; reduces immunosuppressive cell recruitment	De Giglio et al. (2021)
Combretastatin A4 nanodrug + Plerixafor	Murine model	91.3% tumor growth inhibition; reduced lung metastases	Counters therapy-induced SDF-1/CXCR4 surge that can promote metastasis	Jiang et al. (2019)
Plerixafor-functionalized nanomaterials (photothermal/targeted delivery)	Preclinical solid tumors/NSCLC	Enhanced tumor accumulation and photothermal efficacy	CXCR4-targeted homing improves intratumoral delivery	Zhao et al. (2016)
BKT140 (CXCR4 antagonist) ± chemo/RT	NSCLC cell lines and xenografts	↓Proliferation and clonogenicity; delayed tumor growth; improved therapy responses	Potent CXCR4 antagonism complements cytotoxics	Fahham et al. (2012)
IL-24 + CXCR4 antagonism	Preclinical lung cancer	Enhanced anti-metastatic effect	IL-24 dampens CXCR4 signaling (↓AKT/mTOR/HIF-1α)	Panneerselvam et al. (2015)
FX@HP nanocomplex (CXCR4-inhibiting) + anti-PD-L1	Preclinical	↑T-cell infiltration; ↓immunosuppressive cells; improved PD-L1 efficacy	CXCR4 inhibition synergizes with checkpoint blockade	Li et al. (2020)
Peptide R (CXCR4 inhibitor) + immunotherapy context	Preclinical	↓MIC dissemination; restored T-cell cytotoxicity; limited TAM polarization	Targets CD133^+^CXCR4^+^ MICs; reactivates antitumor immunity	D'Alterio et al. (2020)
FM@PFC nanoemulsions (CXCR4 antagonist + anti-STAT3 siRNA)	Preclinical lung metastasis	↓Invasion/angiogenesis/immunosuppression; ↑apoptosis	Pulmonary delivery; dual targeting of CXCR4/STAT3	Li et al. (2019)
Low-dose multi-drug combo (Etoricoxib + Plerixafor + Afatinib + Cabozantinib)	NSCLC PDX	ORR 81%, CBR 100% in therapy-resistant models	Breaks cellular tumorigenic network crosstalk; includes CXCR4 blockade	Li et al. (2025)
Plerixafor targeting stroma	Preclinical	↓Metastasis via impaired stromal recruitment and p38 MAPK activation	Interrupts stromal support of metastasis	D’Alterio et al. (2012)

These liabilities can be countered by adaptive pathways (CXCR7 compensation, STAT3 activation, stromal re-engagement), which tailored combinations, can counteract: chemo/RT (to take advantage of tumor-cell mobilization after transiting protective niches), ICI (to overcome antibodies’s immune homeostasis based on CXCR4), and anti-angiogenic/vascular-disrupting (to suppress CXCL12/CXCR4 rebound of therapy). This framework connects axis biology to rational partnering and sequencing.

Chemoresistance is dictated in significant part by the high activity of CXCR4 in the chemo protection of cancer cells within stromal niches (López-Gil et al., 2021). In lung cancer mouse models, co-administration of plerixafor with cisplatin, paclitaxel, and gemcitabine reduced tumor burden and metastasis vs. chemotherapy alone (Li H. et al., 2021). Jiang et al. showed that CXCR4 expression increases following treatment with vascular-disrupting therapy increases CXCR4 signaling. Combining plerixafor + combretastatin A4 nanodrug produced marked tumor growth inhibition (≈91%) and fewer lung metastases in mice (Jiang et al., 2019). Similarly, Fahham et al. evaluated BKT140, a novel CXCR4 antagonist, in NSCLC cell lines and found that it inhibited proliferation, reduced colony formation, and delayed tumor growth in xenograft models, while also enhancing chemotherapy and radiotherapy responses, demonstrating its potential in combination therapy (Fahham et al., 2012). Panneerselvam et al. further explored the SDF-1/CXCR4 cascade and found that interleukin-24 (IL-24) can inhibit CXCR4 signaling, destabilizing CXCR4 mRNA and reducing downstream AKT, mTOR, and HIF-1α activation, suggesting that IL-24 in combination with CXCR4 antagonists enhances anti-metastatic effects in lung cancer (Panneerselvam et al., 2015).

Beyond its role in chemotherapy, plerixafor has shown promise in overcoming immunotherapy resistance by enhancing T-cell infiltration and reversing the immune-excluded phenotype of tumors. CXCR4-mediated signaling in lung cancer contributes to T-cell exclusion, immune evasion, and recruitment of immunosuppressive cells, which suppress anti-tumor immunity (Martin, 2024). Li et al. developed FX@HP, a CXCR4-inhibiting nanocomplex, which enhanced PD-L1 therapy efficacy by enhancing T-cell infiltration and reducing immunosuppressive cells, indicating that CXCR4 inhibition can remodel the TME to optimize ICI responses (Li et al., 2020). In a related study, Cao et al. revealed that CXCR4 is overexpressed in exhausted CD8+PD-1high T cells, limiting immune response, and demonstrated that blocking CXCR4 restored T-cell function through JAK2-STAT3 inhibition, reinforcing the role of CXCR4 blockade in enhancing immunotherapy (Cao et al., 2024). Similarly, Fortunato et al. identified a subset of CD133+CXCR4+ metastasis-initiating cells (MICs) that contribute to immune suppression and found that Peptide R, a novel CXCR4 inhibitor, effectively reduced MIC dissemination, restored T-cell cytotoxicity, and limited TAM polarization, further supporting the therapeutic potential of CXCR4 blockade in combination with immunotherapy (Cao et al., 2024). Early human evidence exists in other solid tumors, but no randomized data demonstrate added clinical benefit in lung cancer to date.

Radiotherapy remains a cornerstone of lung cancer treatment, yet CXCR4-mediated DNA damage repair mechanisms contribute to radioresistance, allowing tumor cells to survive and repopulate following radiation exposure. Preclinical studies report that plerixafor enhances radiosensitivity by inhibiting CXCR4-linked DNA repair (Eckert et al., 2019; Kim et al., 2021). D’Alterio et al. found that CXCR4 inhibition using plerixafor significantly reduced metastasis by blocking stromal cell recruitment and p38 MAPK activation, impairing tumor cell survival (D’Alterio et al., 2012). In another study, Li et al. developed FM@PFC nanoemulsions containing a CXCR4 antagonist and anti-STAT3 siRNA, showing that pulmonary delivery of these nanoemulsions reduced tumor invasion, angiogenesis, and immunosuppression while inducing apoptosis, demonstrating a novel approach to improving lung metastasis therapy (Li et al., 2019). Additionally, plerixafor has been explored in combination with multi-targeted regimens for therapy-resistant lung cancer (Kast et al., 2022). Gürgen et al. tested a low-dose combination regimen consisting of Etoricoxib, plerixafor, Afatinib, and Cabozantinib in NSCLC patient-derived xenograft (PDX) models, achieving an 81% overall response rate (ORR) and 100% clinical benefit rate (CBR), even in therapy-resistant adenocarcinomas and squamous cell carcinomas lacking targetable mutations, reinforcing the importance of CXCR4 inhibition in overcoming treatment resistance (Gürgen et al., 2022). Reinholdt et al. demonstrated that plerixafor enhances rituximab’s effectiveness in diffuse large B-cell lymphoma (DLBCL) by reducing CXCR4 expression and increasing tumor apoptosis, supporting the potential expansion of CXCR4-targeted therapies beyond lung cancer (Reinholdt et al., 2016). In general, the integration of plerixafor into multimodal treatment strategies has shown great significance potential in lung cancer therapy, especially in combination chemotherapy, immunotherapy, and radiotherapy. CXCR4 inhibition offers a compelling approach to increase lung cancer outcomes through sensitization to therapy, shift immune activation, and mobilization of cancer cells from protective niches as shown in Figure 2.

There is a limited collection of trials involving CXCR4 antagonists in lung cancer. Randomized Phase II 5 of LY2510924 administered as a peptide antagonist on carboplatin/etoposide in extensive disease SCLC (N = 90) failed to improve either PFS or OS compared to chemotherapy alone, but the safety was acceptable. There are a few other investigations that are pre-clinical or pre-imaging feasibility, using [^68^Ga]-based tracers or [^64^Cu]-plerixafor, and are hypothesis-generating and not claim-arming. The probable causes are insufficient biomarker-enriched selection, adaptive compensation, and incompetent scheduling as compared with transient cell mobilization. Contemporary designs predefining the thresholds of CXCR4-positivity and integrating plerixafor with chemo, RT/ICI are expected to fill these gaps.

## 4 The broader therapeutic potential of CXCR4 inhibition

### 4.1 CXCR4 as a target in lung cancer

**FIGURE 3:**
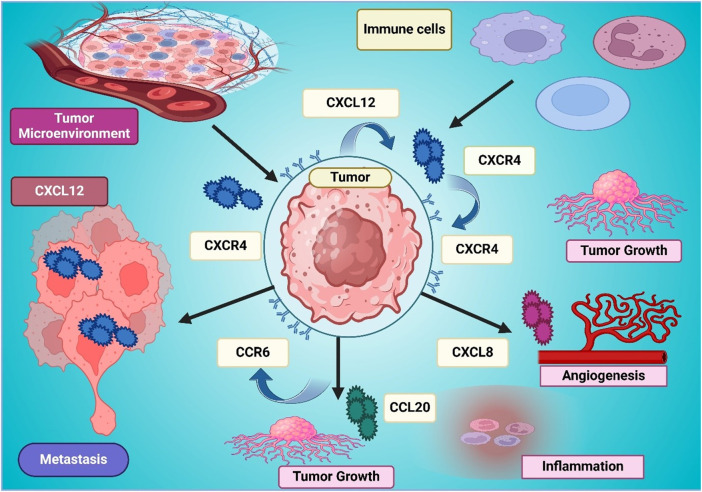
The figure illustrates the role of CXCL12-CXCR4 signaling in the tumor microenvironment, promoting tumor growth, metastasis, angiogenesis, and inflammation. CXCL12 secreted in the tumor microenvironment attracts immune cells and activates CXCR4 on tumor cells, enhancing their proliferation and invasion. CXCL8 and CCR6-CCL20 interactions further contribute to tumor progression by stimulating angiogenesis and inflammation. The interconnected signaling pathways create a supportive niche for tumor survival, emphasizing the significance of CXCR4 as a therapeutic target in cancer treatment.

Over the past years, the CXCR4/CXCL12 cascade has been a significant therapeutic target in lung cancer since it offers a major function in cancer development, metastasis, immunological evasion, and resistance to treatment (Wang et al., 2016; Wald, 2018). Having been shown to overexpress CXCR4, lung cancer is a very strong predictor of poor prognosis, increased metastatic potential, and decreased survival rates, making it a good candidate target for a treatment intervention (Spiro and Porter, 2002). It is through the activation of CXCR4 that the proliferation and survival of tumors are promoted, as well as invasion through oncogenic pathways (Habanjar et al., 2023). Liu et al. demonstrated that hypoxia enhances CXCR4-mediated metastasis via HIF-1α and HIF-2α activation, promoting cancer cell adhesion, movement, and invasion in response to CXCL12, indicating that targeting CXCR4 alongside hypoxia pathways may provide novel therapeutic strategies (Liu et al., 2006). Figure 3 shows CXCL12-CXCR4 signaling facilitating tumor development, metastasis, angiogenesis, inflammation, and immune cell recruitment.

CXCR4 is known to direct lung cancer cells toward CXCL12-rich metastatic sites such as the bone marrow, liver, and brain, fueling metastatic dissemination. Chen et al. found that CXCR4 expression is significantly higher in NSCLC brain metastases, correlating with poorer survival rates, supporting CXCR4 as a key driver of brain-specific metastasis (Chen et al., 2011). Similarly, CXCR4 enhances invasion and migration via EGFR/MMP-9 upregulation (Zuo et al., 2017). Burger et al. further highlighted the role of CXCR4 in SCLC metastasis; CXCR4 promotes invasion/adhesion to bone-marrow stroma; antagonists disrupt this mechanism (Burger et al., 2003).

Beyond metastasis, CXCR4 contributes to immune suppression within the TME. CXCR4 overexpression is correlated with reduced T-cell infiltration, increased MDSCs and Tregs, and resistance to ICIs (Li X. et al., 2021). Cao et al. observed that CXCR4 is overexpressed in exhausted CD8+PD-1high T cells, leading to immune dysfunction and therapy resistance, but CXCR4 inhibition restored T-cell function and enhanced response to ICIs, suggesting CXCR4 blockade as a strategy to improve immunotherapy efficacy (Cao et al., 2024). Wald et al. demonstrated that CXCL12-expressing cancer-associated fibroblasts (CAFs) facilitate cancer progression by supporting CXCR4+ cancer cell survival, reinforcing the significance of targeting the CXCR4/CXCL12 to disrupt tumor-stroma interactions (Wald et al., 2011). In addition to immune evasion, CXCR4 also contributes to chemoresistance and radioresistance by promoting DNA repair mechanisms and survival signaling in lung cancer cells, reducing the efficacy of conventional therapies (Césaire et al., 2022). Jung et al. demonstrated that CXCR4+ cancer stem-like cells drive therapy resistance in NSCLC and that CXCR4 inhibition (AMD3100, siRNA) suppressed sphere formation and tumorigenicity, suggesting that targeting CXCR4+ NSCLC stem-like cells could overcome drug resistance and enhance radiotherapy response (Jung et al., 2013). Similarly, Kim et al. found that CXCR4 enhances radiation resistance via STAT3/Slug signaling and that CXCR4 inhibition sensitized NSCLC cells to ionizing radiation, reinforcing its role in radiotherapy resistance mechanisms (Kim et al., 2021).

Several clinical studies have further validated CXCR4 as a biomarker for lung cancer prognosis. Zhou et al. conducted a meta-analysis of 1,446 NSCLC patients across 13 investigations, finding that CXCR4 expression was significantly associated with advanced-stage disease, metastasis, and reduced survival, reinforcing its role as a prognostic marker (Zhou et al., 2015). Similarly, Otsuka et al. analyzed 170 NSCLC biopsies and found that CXCR4 overexpression in stage IV NSCLC correlated with significantly worse survival, particularly in females, suggesting that CXCR4-targeted therapy may be especially beneficial in advanced lung cancer patients (Otsuka et al., 2011). The role of CXCR4 in lung cancer therapy extends beyond metastasis and resistance, with targeted approaches being actively explored (Ngamcherdtrakul and Yantasee, 2019). Choi et al. demonstrated that CXCR4, but not CXCR7, is essential for CXCL12-mediated metastasis, supporting CXCR4 inhibition as a strategy to prevent NSCLC progression (Choi et al., 2014). Singla et al. found that CXCR4 inhibition slowed metastatic tumor growth in an advanced NSCLC model, reinforcing the potential of CXCR4-targeted therapies in controlling late-stage disease (Singla et al., 2015). Hartmann et al. further identified that CXCR4-driven adhesion enhances chemotherapy resistance in SCLC by activating integrin signaling, suggesting that CXCR4 inhibition could prevent tumor-stroma interactions that contribute to residual disease and relapses (Hartmann et al., 2005).

**FIGURE 4:**
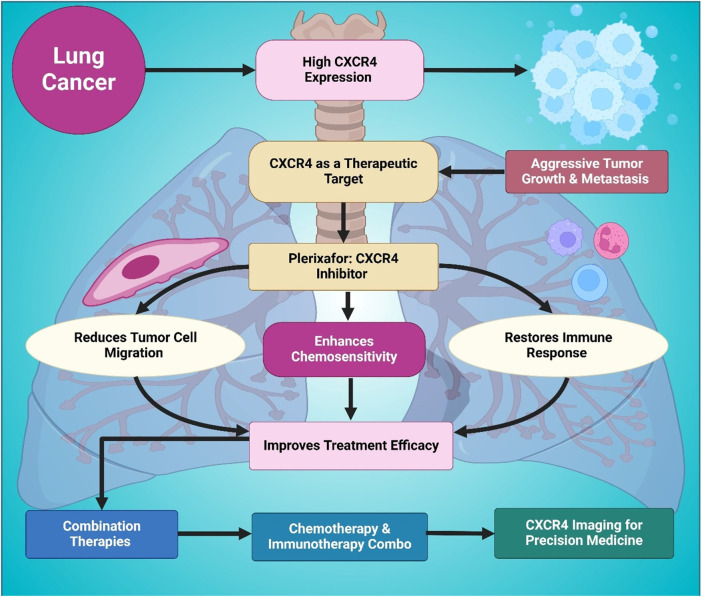
This figure outlines the role of CXCR4 in lung cancer progression and therapy. Elevated CXCR4 expression is associated with aggressive tumor growth and metastasis, making it an important therapeutic target. Usage of the CXCR4 inhibitor Plerixafor reduces tumor cell migration, restores immune function, and enhances chemosensitivity. These effects collectively lead to improved treatment efficacy when combined with chemotherapy or immunotherapy. It also highlights the relevance of CXCR4 imaging for advancing precision medicine in lung cancer management and overcoming therapeutic resistance.

In novel therapeutic approaches, CXCR4-targeted drug delivery strategies have shown promise in overcoming therapy resistance (Costa et al., 2019). Chittasupho et al. developed CXCR4-targeted nanoparticles to enhance doxorubicin delivery to lung cancer cells, finding that CXCR4-specific nanocarriers improved drug accumulation and therapeutic efficacy, demonstrating that CXCR4 inhibition could be used in nanotechnology-driven chemotherapy enhancements (Chittasupho et al., 2014). Li et al. investigated the co-expression of uPAR and CXCR4 in SCLC and found that uPAR+CXCR4+ tumors exhibited greater invasion, migration, and metastatic potential, reinforcing the importance of dual targeting strategies (Li et al., 2014). Similarly, Bi et al. found a strong correlation between lymph node metastasis in NSCLC and the expression of VEGF-C and CXCR4, further validating the role of CXCR4 as a predictive marker for disease progression and therapeutic response (Bi et al., 2017). Together, these studies have identified CXCR4 as a major player in driving lung cancer progression, metastasis, immune suppression, and chemo-resistant disease, and reinforce that it is a target worthy of precision medicine. The direct antagonists of CXCR4, combination therapies, and nanocarrier-based strategies of CXCR4 inhibition could offer great clinical advantages in improving lung cancer treatment as shown in Figure 4 and Table 2.

**TABLE 2:** CXCR4’s role in lung cancer progression and therapy.

Experimental models	Key findings	Main outcomes	Mechanisms	Molecular pathways	Reference
Gefitinib-resistant A549/GR cells	CXCR4+ NSCLC stem-like cells drive resistance	CXCR4 inhibition suppressed sphere formation	STAT3 pathway regulation	mTOR, Akt, STAT3	Jung et al. (2013)
CXCR4 mRNA/surface expression in SCLC	CXCR4 drives metastasis in SCLC	CXCL12 binding induced MAPK activation	CXCR4 antagonist blocks adhesion	MAPK, CXCL12-CXCR4	Burger et al. (2003)
CXCL12 enhanced motility and adhesion	CXCR4 and c-Kit promote SCLC progression	CXCR4 and SCF/c-Kit co-activated Akt	CXCR4 and c-Kit signaling synergy	Akt, p70 S6 kinase	Kijima et al. (2002)
HIF-1α and HIF-2α regulate CXCR4	Hypoxia enhances CXCR4 in NSCLC	RNAi knockdown reduced metastasis	HIF-1α/2α upregulation	HIF, CXCR4, CXCL12	Liu et al. (2006)
Meta-analysis of 1,446 patients	CXCR4 overexpression in NSCLC	CXCR4 correlated with metastasis	Oncogenic overexpression	CXCR4, NSCLC markers	Zhou et al. (2015)
170 NSCLC biopsies analyzed	CXCR4 overexpression in stage IV NSCLC	High CXCR4 linked to poor survival	CXCR4-mediated survival pathways	CXCR4, tumor markers	Otsuka et al. (2011)
CXCR4 levels in 32 patients	CXCR4 and brain-specific metastasis in NSCLC	Higher CXCR4 in metastatic tumors	CXCR4-driven brain metastasis	CXCR4, CNS migration	Chen et al. (2011)
CXCR4 upregulates EGFR and MMP-9	CXCR4 promotes NSCLC invasion	CXCR4 linked to lymph node metastasis	CXCR4-EGFR-MMP9 interaction	EGFR, MMP-9, CXCR4	Zuo et al. (2017)
CXCR4 enhanced adhesion to ECM	CXCR4-driven adhesion in SCLC	Tumor-stroma interactions and chemo-resistance	CXCR4-integrin axis	Integrins, CXCR4	Hartmann et al. (2005)
CXCR4 knockdown abolished migration	CXCR4 but not CXCR7 mediates NSCLC metastasis	CXCR7 had no effect	CXCR4 essential for metastasis	CXCR4, CXCR7, CXCL12	Choi et al. (2014)
CXCR4 siRNA plasmid downregulation	CXCR4 inhibition reduces NSCLC invasion	Reduced migration and invasion	CXCR4 silencing	CXCR4, siRNA	Xie et al. (2014)
CXCL12-expressing CAFs near CXCR4+ tumors	CXCL12/CXCR4 in NSCLC progression	ERK signaling and tumor growth	CXCR4-CAF interaction	ERK, CCL20, CXCL12	Wald et al. (2011)
LFC131-DOX PLGA nanoparticles	CXCR4-targeted nanoparticles in lung cancer	Enhanced CXCR4-mediated drug delivery	CXCR4-mediated internalization	CXCR4, drug transporters	Chittasupho et al. (2014)
NCI-H1299 NSCLC metastasis model	CXCR4 inhibition slows NSCLC metastasis	AMD3100 slowed metastatic growth	CXCR4 blockade in late-stage NSCLC	CXCR4, metastasis pathways	Singla et al. (2015)
CXCR4-overexpressing A549/GR cells	CXCR4 enhances radiation resistance	STAT3/Slug signaling in IR resistance	CXCR4-STAT3-IR signaling	STAT3, Slug, IR resistance	Kim et al. (2021)
50 SCLC tissue samples analyzed	uPAR and CXCR4 predict worse SCLC prognosis	High uPAR+CXCR4+ linked to metastasis	CXCR4/uPAR synergy	uPAR, CXCR4	Li et al. (2014)
110 NSCLC samples analyzed	CXCR4 and VEGF-C in NSCLC lymph node metastasis	CXCR4 and VEGF-C upregulated	CXCR4-VEGF-C axis	VEGF-C, CXCR4	Bi et al. (2017)

## 5 Challenges and future perspectives

### 5.1 Limitations of CXCR4-Targeted therapy

Despite the promise of CXCR4-targeted therapies in lung cancer treatment, numerous obstacles and limitations must be overcome to enable such therapies to become a part of clinical practice (Ashrafi et al., 2022). Specificity and safety, mechanisms of resistance, and complexities of TME fall in this range of limitations (Khalaf et al., 2021). The major issue with CXCR4-targeted therapies is how to achieve specificity for tumor cells. However, CXCR4 is expressed widely in various normal tissues, notably bone marrow, spleen, and liver, and regulates the function of immune cell trafficking and healthy tissue homeostasis (Tilsed et al., 2022). Thus, the use of CXCR4 inhibitors may result in unwanted side effects, which include excessive immune cell depletion, tissue destruction, or bone marrow suppression, increasing the occurrence of infections or hematologic toxicity (Vasan et al., 2019). A major hurdle to the clinical development of selective CXCR4 antagonists is the need to find those that will target tumor cells without deleterious effects on normal tissues (Ho et al., 2020).

Like many targeted therapies, resistance to CXCR4 inhibitors can occur with time, and in particular, if the inhibitor is combined with other treatments (Roma-Rodrigues et al., 2019). If these chemokine receptors happen to be upregulated on the tumors, tumor cells can continue to migrate and metastasize, even if CXCR4 is inhibited (Kim et al., 2008). Besides, resistance to therapeutic agents results from CXCR4 or its downstream signaling pathway mutations (Korbecki et al., 2022). Further research on the mechanisms of resistance is needed to design strategies that will overcome or delay the resistance from CXCR4-targeted therapies to improve long-term efficacy (Alsayed et al., 2022). The determination of both the success of CXCR4 targeted therapy and the clinical significance of CXCR4 is determined by the TME (Yin et al., 2019). The TME containing extracellular matrix constituents, stromal cells, and immune cells can affect the efficacy of treatment (Kim et al., 2008). As Tregs and MDSCs are immunosuppressive cells that produce barriers to effective immune responses (He et al., 2019), CXCR4 inhibitor enhancement of anti-tumor immunity is restricted (Popper, 2016). In addition, the CXCR4 inhibitors may be partially constrained by the inability to access their targets in hypoxic conditions and chemoresistant niches within the TME, hence reducing their therapeutic capability (Kiefer and Siekmann, 2011).

### 5.2 CXCR4 in personalized medicine

CXCR4 is an important biomarker in lung cancer because of its important molecular marker and target for personalized medicine (Rosell et al., 2013). Therefore, CXCR4 targeted therapies are a promising approach to improve treatment outcomes with a benefit-to-risk ratio as high as possible. Personalized approaches that incorporate CXCR4 status into treatment decisions could enhance patient selection for targeted therapies (Chai et al., 2021; Nengroo et al., 2022a). Guo et al. have shown that CXCR4 overexpression correlated with poor prognosis in NSCLC but was also correlated with a higher response rate to immunotherapy, indicating that CXCR4 expression could serve as a predictive biomarker for immunotherapy efficacy (Guo et al., 2023). Yue et al. further supported this by identifying CXCR4, CXCR5, and CCR7 as prognostic biomarkers in early-stage NSCLC, where CXCR4 and CXCR5 were associated with worse five-year DFS and OS (Yue et al., 2020). This highlights the potential of CXCR4 expression profiling in guiding patient-specific treatment regimens (Nengroo et al., 2022a). Incorporating CXCR4 inhibitors such as plerixafor into combination treatment strategies has also been explored (Srivastava et al., 2022). Given the association between CXCR4 and immune evasion, Mao et al. found that B7-H1 and B7-H3 overexpression in NSCLC tumors correlated with CXCR4-driven immune suppression, while B7-H3 knockdown enhanced T-cell activation and reduced CXCR4 expression, indicating that CXCR4 blockade in combination with ICIs could improve patient responses (Mao et al., 2014). Additionally, Naz et al. reported that Abemaciclib, a CDK4/6 inhibitor, increases radiosensitivity in NSCLC by disrupting DNA repair and metabolic pathways, supporting the concept of CXCR4-targeted therapies in biomarker-selected patients undergoing radiotherapy (Naz et al., 2018).

Beyond direct therapeutic targeting, CXCR4 expression can evolve throughout treatment, necessitating adaptive monitoring strategies (Liu et al., 2024). Marquardt et al. identified CXCR4 expression as a marker of immune-enriched tumors with CD8^+^ T-cell infiltration, while fibroblast activation protein (FAP) overexpression was linked to angiogenesis. These findings suggest that CXCR4-and FAP-targeted PET imaging could serve as a non-invasive tool for personalized therapy selection (Marquardt et al., 2023; Wen et al., 2023). Similarly, Bertolini et al. found that cisplatin treatment in NSCLC increased CXCR4+ MICs and CXCL12 levels, creating a pro-metastatic TME. However, CXCR4 inhibition prevented chemotherapy-induced metastasis, reinforcing the need for biomarker-driven treatment adjustments to mitigate resistance (Bertolini et al., 2021).

Economic considerations also play a role in personalized CXCR4-targeted therapy implementation (Abdollahi et al., 2024). Rudakova et al. examined the cost-effectiveness of Gefitinib as a second-line NSCLC therapy, demonstrating that it increased life expectancy by 6 months compared to Pemetrexed while reducing overall medical costs, suggesting that CXCR4-positive patients may also benefit from economic modeling in treatment selection (Rudakova et al., 2015). Additionally, genomic profiling can refine personalized treatment strategies by identifying genetic variants that influence CXCR4 signaling and therapy resistance (Li X. et al., 2022; Almawash, 2025). Islam et al. investigated functional polymorphisms in the ACRBP gene, revealing deleterious coding variants that may contribute to tumor progression, though further studies are needed to determine their role in lung cancer pathogenesis (Islam, 2022). This aligns with the growing emphasis on genomic-guided therapy adjustments, where ongoing biomarker monitoring helps optimize treatment plans over time (Tashkandi and Younes, 2024). In integrated medicine for lung cancer, CXCR4-targeted therapies can be used to improve therapeutic benefits with reduced unnecessary toxicity. Utilizing CXCR4 as a prognostic and predictive biomarker, treatment regimens can be individualized to enhance survival rate, therapy response, and cost-effectiveness. Genomic analysis of disease and the use of CXCR4-based imaging, immune profiling, and other precision medicine will further improve the medical approach to the treatment of patients who will receive the most efficacious treatment of their disease in the form that is most tailored to their needs.

Since there is inter- and intra-tumor variability, a combination of CXCR4-PET and tissue/blood biomarkers is a viable enrichment approach to trials and practice. Since the CXCR4-directed PET/CT is feasible in lung lesions and trials indicate radiolabeled plerixafor agents, prespecification, including (i) a positivity rule, (ii) treatment of lesion-level heterogeneity, and (iii) on-treatment PET at a specified fixed time point with a pre-determined percent-change threshold, should indicate pharmacodynamic target engagement versus non-engagement. The readout based on tissue IHC and blood can be overlaid to prove baseline positivity and follow the dynamics in parallel with PET. These working regulations conform to the applicability of biomarkers to trial admission and response measurement and capitalize on the present PET/theranostic framework.

### 5.3 Clinical-trials landscape of CXCR4 inhibition

CXCR4 antagonists have a limited clinical testing experience in lung cancer. The most recent randomized trial of efficacy (only in lung cancer) LY2510924 (peptide CXCR4 antagonist) combined with carboplatin/etoposide in ED-SCLC N = 90 randomized phase II, with no observed improvement in PFS/OS over chemotherapy alone, although safety was acceptable, and CXCR4 IHC was an exploratory biomarker evaluated. This highlights the necessity of biomarker-informed selection and combinatory-refinement strategy dominating in trial sequences in the future (Salgia et al., 2017). Simultaneously, CXCR4-based imaging (e.g., [^68^Ga pentixafor PET/CT]) and [^64^Cu pleixafor PET]) imaging can be done in lung cancer and other solid tumours, providing a strategy to enrich in CXCR4-high disease and to measure on-treatment target engagement (Lapa et al., 2016; Burke et al., 2020).

## 6 Conclusion and future perspectives

Emerging evidence suggests that CXCR4 inhibitors, such as plerixafor, can effectively disrupt tumor-stroma interactions, enhance immune infiltration, and sensitize tumors. Preclinical and early clinical studies indicate that CXCR4 blockade may limit metastatic dissemination, overcome resistance to therapy, and improve overall patient outcomes. However, despite these encouraging findings, several challenges must be addressed before CXCR4-targeted therapies can be fully integrated into clinical practice. Compensatory survival mechanisms associated with the activation of CXCR4 inhibition may limit the therapeutic efficacy. CXCR4 blockade could trigger alternate signaling pathways such as PDGFRB, STAT3, as well as CXCR7, which may initiate tumor progression and therapy resistance. Furthermore, patient heterogeneity due to tumor heterogeneity and changeable CXCR4 expression makes it difficult to find the best patient group for the CXCR4-targeted therapies. To maximize clinical efficacy while minimizing unnecessary toxicity, integrating CXCR4 expression profiling with immune landscape characterization and genomic analysis will be essential for the biomarker-driven treatment selection.

As the complexity of tumor progression driven by CXCR4 is so great, combination therapies have enormous promise in improving the treatment outcome. In preclinical studies, the combination of ICIs, chemotherapy or radiotherapy, and CXCR4 inhibitors has shown synergistic effects and (reliable) proof of concept, but the most effective dosing regimens, sequencing strategies, and combination partners need further clinical validation. Further, CXCR4 targeted approaches for drug delivery, i.e., nanomedicine-based formulation, antibody-drug conjugate, and CXCR4 inhibitor radionuclide, may further enhance tumor selectivity and therapeutic efficacy while decreasing the off-target effects. Similar to the CXCR4 antagonists, advances in CXCR4-PET imaging enable a non-invasive method to measure CXCR4 expression in tumors for real-time adjustment of treatment and individual patient-tailored therapy modifications based on individual patient response. Its involvement in CNS metastases in patients with lung cancer is further established. Penetration of many CXCR4 targeted agents into the brain is limited by the BBB, hence, the development of brain penetrant CXCR4 inhibitors or alternative delivery, such as Intrathecal, is important.

Definitive clinical benefit in lung cancer has not yet been demonstrated; ongoing and future phase II/III trials with CXCR4 imaging/biomarker-guided selection will be essential to determine where plerixafor adds value. Future research should focus on addressing the action of CXCR4 inhibition to CNS-specific metastases in patients with advanced-stage lung cancer and treatment-resistant brain metastases. CXCR4 targeted therapies promise is great, however, long-term safety needs to be carefully reviewed. CXCR4 has an important role in hematopoiesis as well as in immune cell trafficking and normal stem cell function, which could lead to concerns regarding chronic inhibition of immune homeostasis and normal tissue function. A long-term toxicity assessment, potential immune-related side effects, and optimal dosing schedule need to be put in future trials to provide sustained therapeutic benefits with no complications.

Up to now, the clinical benefit of CXCR4 antagonism has not been demonstrated in any randomized trial in patients with lung cancer; phase II/III trials that are biomarker-enriched and imaging-guided are required to determine the points of value addition of plerixafor.
